# Herbst’s Method

**Published:** 1886-01

**Authors:** 


### EDITORIAL.

### Herbst's Method—Boedecker.

A few days ago we had the opportunity and privilege of spending a few hours with Dr. C. F. Boedecker, of New York, when the opportunity was afforded, to witness an operation according to Dr. Herbst's method of filling. Dr. Boedecker is more familiar with this method of filling than any other person in this country, and it was a great matter of interest to witness the operation. It was in an approximate filling of the superior lateral incisor, the cavity that is ordinarily regarded as rather difficult to fill. The cavity was prepared much in the ordinary method; the gold was introduced and placed in position by the ordinary filling instrument, then condensed with the appropriate Herbst instrument. The manipulation of Dr. Boedecker seemed very simple and easy; this, however, was most probably the result of his knowledge of the method, and his experience in its use. Some I know have attempted to operate merely from the descriptions that have been made, and have failed. The result of this operation was a complete success. The gold used, was prepared expressly for the purpose, and has some qualities that are not possessed by the ordinary gold foil, or indeed, by any other, so far as I know. Upon witnessing this operation, our appreciation of its practicability was very much enhanced. It is questionable whether any one should attempt to practice this method without special instruction or assistance; many valuable things, by these means, have been brought in disrepute, and have failed to serve the purpose which they might have done under different circumstances. It should be a rule, that every practitioner should have the best information attainable in regard to every new process and method before attempting to adopt it.

